# Comparison of Electronic Data Capture (EDC) with the Standard Data Capture Method for Clinical Trial Data

**DOI:** 10.1371/journal.pone.0025348

**Published:** 2011-09-23

**Authors:** Brigitte Walther, Safayet Hossin, John Townend, Neil Abernethy, David Parker, David Jeffries

**Affiliations:** 1 Statistics and Data Management Department, Medical Research Council, Fajara, The Gambia; 2 Department of Bio-Informatics, University of Washington, Seattle, Washington, United States of America; 3 Population Services International (PSI), Washington, D. C., United States of America; University of Modena and Reggio Emilia, Italy

## Abstract

**Background:**

Traditionally, clinical research studies rely on collecting data with case report forms, which are subsequently entered into a database to create electronic records. Although well established, this method is time-consuming and error-prone. This study compares four electronic data capture (EDC) methods with the conventional approach with respect to duration of data capture and accuracy. It was performed in a West African setting, where clinical trials involve data collection from urban, rural and often remote locations.

**Methodology/Principal Findings:**

Three types of commonly available EDC tools were assessed in face-to-face interviews; netbook, PDA, and tablet PC. EDC performance during telephone interviews via mobile phone was evaluated as a fourth method. The Graeco Latin square study design allowed comparison of all four methods to standard paper-based recording followed by data double entry while controlling simultaneously for possible confounding factors such as interview order, interviewer and interviewee. Over a study period of three weeks the error rates decreased considerably for all EDC methods. In the last week of the study the data accuracy for the netbook (5.1%, CI95%: 3.5–7.2%) and the tablet PC (5.2%, CI95%: 3.7–7.4%) was not significantly different from the accuracy of the conventional paper-based method (3.6%, CI95%: 2.2–5.5%), but error rates for the PDA (7.9%, CI95%: 6.0–10.5%) and telephone (6.3%, CI95% 4.6–8.6%) remained significantly higher. While EDC-interviews take slightly longer, data become readily available after download, making EDC more time effective. Free text and date fields were associated with higher error rates than numerical, single select and skip fields.

**Conclusions:**

EDC solutions have the potential to produce similar data accuracy compared to paper-based methods. Given the considerable reduction in the time from data collection to database lock, EDC holds the promise to reduce research-associated costs. However, the successful implementation of EDC requires adjustment of work processes and reallocation of resources.

## Introduction

Conventional data collection for clinical and scientific trials has focused on paper-based case report forms (CRF) followed by double data entry into a relational database. Recent technological advances and considerable reduction in prices for portable computers make EDC an intriguing alternative. The major advantages of EDC would be the ability to enter, review and analyse data in real-time and to implement online data validation checks to assure data quality more effectively at the point of entry. Regulatory bodies in the USA and Europe address data protection and privacy, electronic data interchange and the use of computerized systems in clinical trials in their regulations and directives [1], [2], [3], [4]. Title 21 CFR Part 11 of the Code of Federal Regulations [5] deals with the FDA guidelines on electronic records and electronic signature in the United States. It defines the criteria under which electronic records and electronic signatures are considered to be trustworthy, reliable and equivalent to paper records. It requires implementation of controls, including system validations, audit trails, electronic signatures, and documentation for software and systems involved in processing electronic data to ensure the authenticity, integrity, and the confidentiality of electronic records.

In fact, as evidenced by a considerable body of literature, the use of portable handheld computer technology in the field of health care and clinical research is on the rise [6]. However, most of the literature is descriptive, focusing on the technology, the methods and/or the experience. Descriptive studies that often lack a control group are limited with regard to comparisons of the effectiveness of different methods. A review of randomized controlled trials comparing the effectiveness of hand held computers with paper methods from 2006 summarizes that handheld computers appear superior in timeliness of receipt and data handling, but that the studies reviewed used different study designs and error definitions for assessment of data accuracy and reported inconsistent results [7].

This paper compares the performance of four electronic data capture methods (see supplementary material, File S1, for machine specifications) with the performance of conventional paper-based data collection in a Gambian medical research field station setting:

- PDA (pen operated)
- Netbook (a small, lightweight laptop computer with reduced computing power)
- Tablet PC (a small laptop computer, which is equipped with a touch screen), and
- EDC during a telephone interview via mobile phone

There are a plethora of devices suitable for EDC; this study uses three technology types, netbook, tablet PC and PDA , which are commonly used for EDC. Scanning devices such as smartpen or optical character recognition technologies [8], [9], were not considered in this study. The objectives of the study were to combine a formal comparison with a demonstration that EDC is viable for clinical trials routinely undertaken at The MRC Gambia. Tools to improve EDC data accuracy, automated range and consistency checks or skip patterns, were not evaluated. Data entry during telephone interviews, in which CRFs were administered via mobile phone, was included due to the high and increasing number of mobile subscribers (In 2009, 800,000 out of a population of 1.7 million) in The Gambia.

Although the study was primarily powered to compare error rates between the five data capture methods, secondary analysis was performed analysing response times and investigating if any training effects (improvement of data accuracy and/or duration of data capture from the first to the third week of the study) could be measured. The acceptability of EDC among the interviewers was qualitatively evaluated.

## Methods

The study, which was approved by the Joint Gambian Government / MRC Ethics Committee, was designed as a 5 by 5 Graeco Latin square to compare error rates between the five data entry methods. A Graeco Latin square is an efficient design allowing differences to be simultaneously adjusted for three possible sources of confounding: interviewer, interviewee and order of interview, as depicted in Table 1. Based on an error rate estimate of 5% for the standard CRF based data collection method within The MRC Gambia, the square was randomly replicated three times to obtain 80% power to detect a significant difference at the 5% level between the five methods. Blinded from the interviewer, each of the 15 interviewees (5×3 replications) was given one unique CRF with randomly generated answers, which s/he was tasked to read aloud in response to the questions the interviewers would asked during the interviews. The randomly generated answers served as the gold standard to assess data accuracy for all five methods including the conventional method [10].

**Table 1:** Schematic of the Graeco Latin Square Design.

	S1	S2	S3	S4	S5	
**F1**	M1 O1	M2 O4	M3 O2	M4 O5	M5 O3	**Day1**
**F2**	M2 O2	M3 O5	M4 O3	M5 O1	M1 O4	**Day2**
**F3**	M3 O3	M4 O1	M5 O4	M1 O2	M2 O5	**Day3**
**F4**	M4 O4	M5 O2	M1 O5	M2 O3	M3 O1	**Day4**
**F5**	M5 O5	M1 O3	M2 O1	M3 O4	M4 O2	**Day5**

Interviewer/Fieldworker 1–5 (F1–F5).

Interviewee 1–5 (S1–S5).

Method 1 (M1): face-to-face interview & MRC standard paper-based data capturing and processing.

Method 2 (M2): face-to-face interview & EDC using a netbook.

Method 3 (M3): face-to-face interview & EDC using a tablet-PC.

Method 4 (M4): face-to-face interview & EDC using a PDA.

Method 5 (M5): telephone interview & EDC using a laptop.

Order (O1–O5): Each interviewer/field worker interviewed 5 interviewees/volunteers per day. O1–O5 indicate the order in which the interviews were conducted (O1: 1st interview at a particular day, O2: 2nd interview, O3: 3rd interview, etc.).

Within the MRC Gambia research structure, field workers and nurses are responsible for administering CRFs in the community and would be the staff to pioneer the use of EDC tools in the future. Consequently the five interviewers were randomly selected from a pool of 12 available field workers and nurses. The fifteen interviewees were voluntarily and randomly recruited from 400 MRC staff based at the main Fajara site.

The CRF for this study was a facsimile of typical CRFs from the range of clinical and research studies undertaken at the MRC. It was developed in the style of the World Health Organization's individual questionnaire, which was used for the World Health Survey in 2002 [11].To make the interviews as authentic as possible, genuine questions flowed through identifier, demographic and adult/child clinical data sections, but with an emphasis on the question fields, free text and date fields, which are typically associated with the highest error rates. The number of children per interviewee in the scripted responses was restricted to four to ensure the comparability between interviews regarding the number of fields and duration of the interview.

For clinical trials conducted at the MRC, OpenClinica® [12], an open source software package exclusively designed for EDC and compliant with Good Clinical Practice (GCP) requirements, is the software of choice. OpenClinica® is a web-based application ideally suited to wireless client-server implementation and is available for free or as paid edition, which includes product support.

MRC field workers have little or no professional experience with handheld devices and a wide range of informal computer experience, largely dependent on their age. They are not routinely trained in electronic data entry techniques or the use of data management tools such as OpenClinica®. Consequently the five randomly selected field workers (plus one reserve) were given a three day training course typically offered to data entry personnel at the MRC in the Gambia. The major areas covered included an introduction to using the OpenClinica® software and the web based application for EDC, familiarization with the electronic devices and interview practice.

In an effort to create realistic field conditions to test the performance of the screens and ruggedness of the EDC machines, the interviews were conducted outside in a tree shaded area on the MRC Fajara field station. Telephone interviews were conducted indoors with the interviewer sitting next to a conference call facility. The interviewee was called on her/his mobile phone and data were simultaneously entered into a PostgreSQL database using OpenClinica® as front end on a standard laptop. The control method was the conventional paper-based CRF with adjudicated double entry into OpenClinica®, by two out of a possible 25 randomly selected MRC data entry staff.

The interviewers were asked to record the start and end time of the interview process, excluding the time needed for starting the program and entering their credentials.

OpenClinica® was not used in PDA as no compatible version for PDA was released at the time of the study. Therefore, ASP.NET technology for mobile devices was used with SQL Server at the backend to develop an application program to capture data using PDA device. Nevertheless, the underlying application architecture was client-server based for both OpenClinica® (Version 3.0.2) and ASP.NET with wireless connectivity to communicate with the server. Wireless technology to rural areas, where many field studies take place, is in early stages of development within the Gambia [13], [14], [15], [16], [17], therefore this approach was not used for the study. The netbook and the tablet PC each had sufficient memory to store the requisite data on the device. With the PDA, conventional data capture, and telephone interview modalities, data were remotely entered into a database on SQL server 2000. To prevent data loss the databases were backed up daily. The five resulting databases were compared to the gold standard database of the randomly generated answers using a generic web-based double entry application for SQL server databases [18]. Any missing values or inconsistencies with exception of the use of capital and lower case letters counted as an error.

Assuming salary costs of US $ X/hour for a data entry clerk, US $ 1.33*X/hour for a field worker, US $ 1.67*X/hour for a data supervisor and US $ 2.33*X/hour for a data manager, salary costs per correctly entered field were calculated with the following formulas:

For the standard method (involvement of a field worker, a data entry clerk and a supervisor):
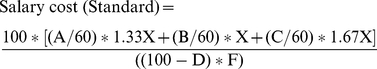
For EDC methods (involvement of a field worker and a data manager):
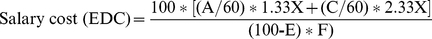

- A = Interview duration
- B = Duration of double entry of the data
- C = Time needed for synchronization of data bases, performance of range and consistencychecks
- D = Error rate for the standard method (%)
- E = EDC error rate (%)
- F = Total number of data fields per questionnaire

All study volunteers provided informed, written consent to participate in this study.

### Statistics

The first step in our analysis included comparisons of proportions and Wilcoxon signed-rank tests to compare error rates and duration of the interviews between the five data capture methods. In a second analysis step logistic mixed effects models adjusting for confounding effects of ‘interviewer’ were fitted to compare the overall error rates and the error rates for each field type: text, date, numerical, single select, and skip (separately). The two possible confounders ‘interviewee’ and ‘order’ were not included in the models since they did not prove to be significant at the 5% level (two sided). To compare the speed of the interviews using EDC methods with the standard paper-based method, a mixed effect model adjusting for random effects for the 25 combinations of fieldworker and device was fitted to the square root of the duration of the interview. All models included week and order as explanatory variables. Where appropriate, interaction terms for device/week and order/week were included as well. The analyses were performed using the statistical software Stata (StataCorp. 2009. Stata Statistical Software: Release 11. College Station, TX: StataCorp LP). P_adjusted_ in the results section and the tables refers to p-values, which had been adjusted for confounding factors.

## Results

Five interviewers, one female and four male with the age of 29, 38, 42, 45, and 46, respectively, were randomly selected to conduct 15 interviews each. Three were field workers and two nurses by profession. Their MRC work experience ranged from two to 25 years (median 14 years) and their experience as fieldworker from zero to 25 years (median 14 years).

Each questionnaire comprised of 115 questions. Due to problems with the partial date function in OpenClinica® (a known bug in OpenClinica® version 3.0.2), which led to inconsistent swaps of the day and month fields within a date during the data extraction process, only 111 fields per CRF were analyzed; 25 (22.5%) text, 10 (9.0%) date, 6 (5.4%) numerical, 37 (33.3%) single select and 33 (29.7%) skip questions. 3 (9.1%) of the skip questions were text fields , 9 (27.3%) numerical, and 21 (63.6%) single select fields.

73 (97.3%) of the 75 interviews were conducted according to the time schedule. Two interviews using the tablet PC could not be conducted due to problems with the software during the first study week. One record in the PDA database was partially deleted immediately after the information had been collected. The time record for this interview could not be retrieved. In total 8,103 (73*111) data fields were analysed to calculate error rates. The information, which was collected with the PDA, but was erroneously deleted, was counted as missing values.

(For the access database holding the generated reference and the raw data from this study see supplementary material, File S2).

### Error rates

#### Overall error rates – Error rates decreased significantly over time for all methods, except the standard and EDC using the tablet PC - In the last study week the error rates for the netbook and the tablet PC were not significantly different from the error rates of the standard method

**Figure 1:**
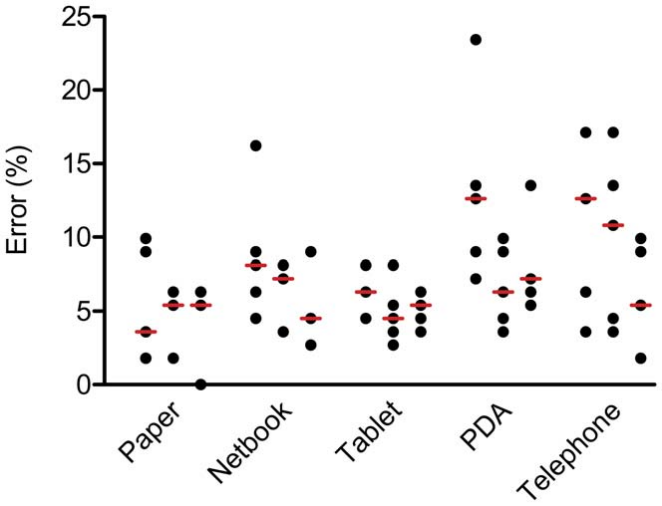
Error rates (%) per questionnaire. The graph above presents the error rates (%) per interview for the standard paper-based data collection method and the four electronic data capture methods; netbook, tablet PC, PDA, and telephone interview in combination with EDC, in the 1^st^, 2^nd^, and 3^rd^ study week. The bars represent median error rates per method and week.

The error rates for each individual interview as well as median error rates per week and device are presented in figure 1.

In the first study week the lowest error rates (%) - defined as number of errors per 100 fields - were found for the standard data capturing method, followed by the EDC methods using the tablet PC and the netbook (table 2). The highest error rates were measured for the EDC method using the PDA and the telephone interviews. Over the study period of three weeks the error rates improved significantly for all EDC methods except for the tablet PC, which was the EDC method with the lowest error rate in the first week (netbook: −43.2%, PDA: −40.2%, telephone: −40.0%, and tablet PC: −17.5%). For the entire study period the error rates measured for EDC using the tablet PC were not significantly different compared to the standard data collection on paper (1^st^ week: p_adjusted_ = 0.55, 2^nd^ week: p_adjusted_ = 0.68, and 3^rd^ week: p_adjusted_ = 0.20). The netbook error rate was significantly higher in the first study week compared to the standard method (p_adjusted_ = 0.019), but in the third week the error rate had dropped to a level which was not significantly different from the error rate measured for the standard method (p_adjusted_ = 0.26).

**Table 2:** Error rate (%) per week - Trend over time.

Method	1st week		2nd week		3rd week		3^rd^ week compared to 1^st^ week	Trend over time
	Mean	CI95%*	Mean	CI95%*	Mean	CI95%*	p**	p_adjusted_ ***
**Paper**	5.2	3.4–7.1	4.3	2.6–6.0	3.6	2.0–5.2	0.189	0.210
**Netbook**	8.8	6.5–11.2	5.9	4.0–7.9	5.0	3.2–6.9	0.013	0.012
**Tablet**	6.3#	3.7–8.9	4.9	3.1–6.7	5.2	3.4–7.1	0.499	0.642
**PDA**	13.2 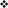	10.3–16.0	6.7	4.6–8.7	7.9	5.7–10.2	0.005	0.003
**Telephone**	10.5	7.9–13.0	9.9	7.4–12.4	6.3	4.3–8.3	0.013	0.014

* Wilson 95% confidence interval for the error rate.

# Error rate(%) for three interviews using the tablet.

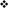
Error rate(%) for four interviews using the PDA.

**p-value for the test of proportions comparing the error rates for the first and third week of the study.

***p-value, mixed effect model adjusting for the clustering effects for ‘fieldworker’ and ‘order’ in which the interviews were conducted.

**Figure 2:**
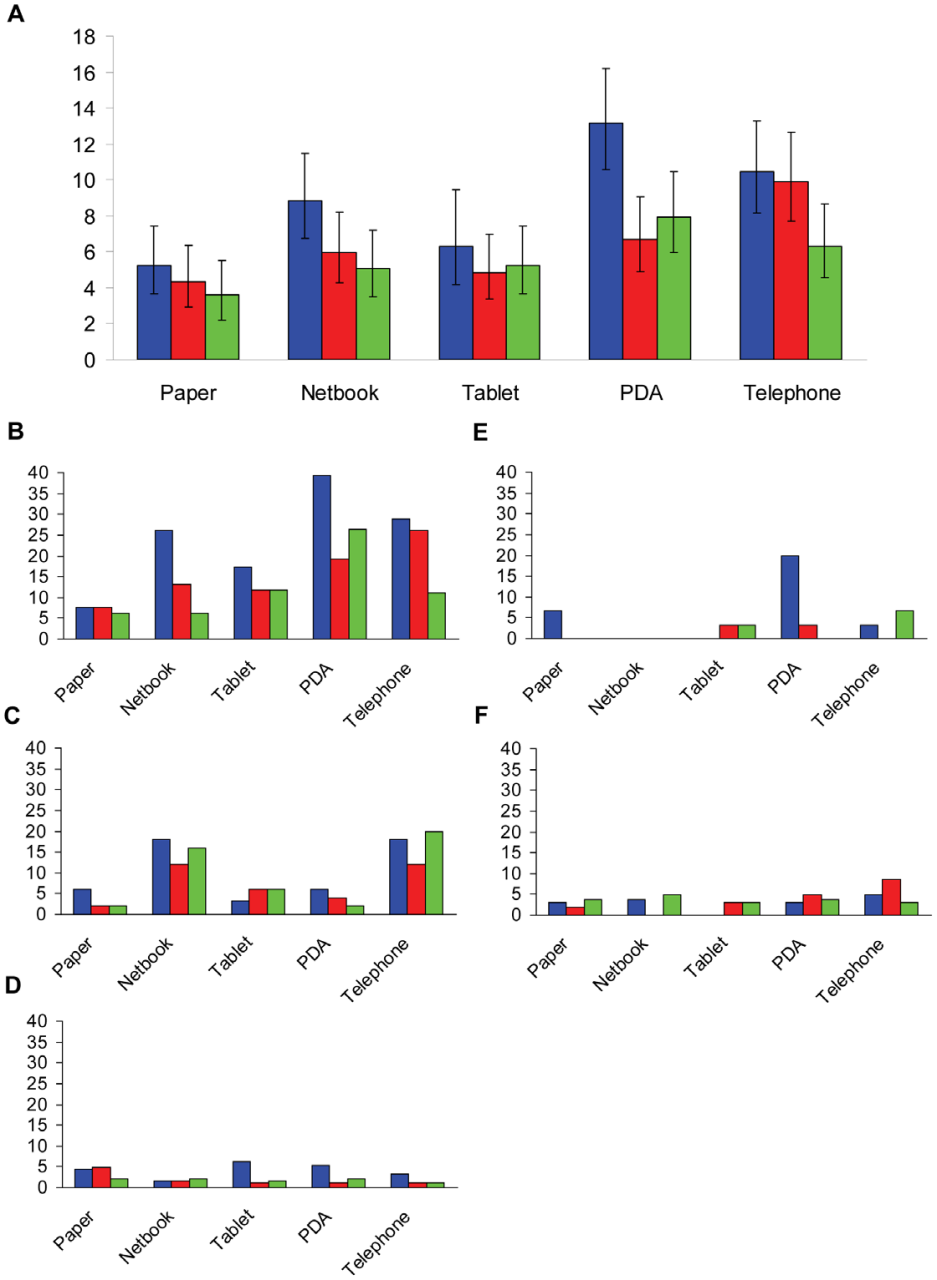
Error rate (%). The overall error rates per 100 fields (%) and 95% confidence Intervals (Wilson) for the standard paper-based as well as the electronic data capturing methods in the first, second and third week of the study (1st week: blue, 2nd week: red, and 3rd week: green) are presented in graph A. The five smaller graphs present the error rate by field type and study week (B: text fields, C: date, D: single select, E: numerical, F: skip).

The presentation of the overall error rate was strongly associated with the type of the question. Therefore error rates were analysed separately for each question category. Mean overall error rates as well as mean error rates per category and week are presented in figure 2.

#### Text - This field type was associated with the highest error rates

Similar to the overall error rate, the lowest error rates for text fields were measured for the standard method and the tablet PC at the beginning of the study (8.8% and 17.3%, respectively). The text field error rates by the third week had improved for all methods including the standard method, with this being significant for the netbook, the PDA, and the telephone interview (p_adjusted_<0.0001, p_adjusted_ = 0.032 , and p_adjusted_ = 0.005). The text field error rate for the netbook decreased steeply from 24.8% in the first week to 6.4% in the third week, which was below, but not significantly different from the error rate of 7.2% which was measured for text fields using the standard method (p_adjusted_ = 0.15). At the end of the study period the text field error rates of 12.8% for the telephone interview and 13.6% for the tablet PC, were found to be significantly higher compared to the standard method (p = 0.011 and 0.025, respectively). At the end of the study the highest error rate for EDC was 26.4% using the PDA.

#### Date - Second-highest error rates were measured for this field type

This study did not detect any significant changes in the error rate for date fields over the duration of three weeks. The error rates for date fields measured for the entire study period were lowest for the standard method, the tablet PC and the PDA, being 5/150 (3.3%, CI_95%_: 1.4%–7.6%) for the paper-based method, 7/130 (5.4%, CI_95%_: 2.6%–10.7%) for EDC using the tablet, and 6/150 (4.0%, CI_95%_: 1.8%–8.5%) for EDC using the PDA. The netbook and the telephone interview had significantly higher error rates with 23/150 (15.3%, CI_95%_: 10.4%–22.0%) and 25/150 (16.7%, CI_95%_: 11.6%–23.4%). Interestingly, for both methods associated with the highest error rates for dates, the data were entered using similar devices (a netbook and a laptop) with keyboards of comparable sizes and OpenCinica® at the front end.

#### Single select - This field type was associated with low error rates - No significant difference in accuracy between the data capture methods was detected

Compared to the error rates for text and date fields, the error rates for questions with answers of single select type were lower, ranging between a minimum of 1.1% and a maximum of 6.3%. This study was not sufficiently powered to detect such small difference as significant. Despite a trend to lower error rates towards the end of the study period for all methods other than the netbook, this study could not show a significant decrease in the error rates for single select questions.

#### Numerical - Lowest overall error rates

Questions with numeric responses comprised the smallest number (5.4%) of all question types. Error rates for this type of questions were uniformly low and ranged from 0% (CI_95%_: 0%–4.1%) for the netbook to 7.8% (CI_95%_: 3.8%–15.2%) for the PDA. The highest error rate of 20% (6/30) was measured during the first study week and can be explained by missing values for numeric fields due to the partial deletion of one record. With 90 observations per device for each method (except the tablet PC with 78 observations, 12 missing observations for numerical values due to the 2 interviews which could not be conducted) this study did not detect any significant trends over time or differences between the individual data capturing methods for numerical fields.

#### Skip - Field type with low error rates - Accuracy did not change over time - No significant difference between data capture methods

Error rates for skip questions did not change significantly over the three weeks period. Lowest error rates for this type of question were found for the EDC method using the tablet PC (2.3%, CI_95%_: 1.3%–4.2%) and the standard paper-based method (2.8%, CI_95%_: 1.7%–4.7%). The error rate for EDC methods using the netbook or the PDA were 3.8% (CI_95%_: 2.5%–5.9%). The highest error rate for skip questions of 5.5% (CI_95%_: 3.8%–7.8%) was measured for the telephone interview, which was slightly and borderline significantly higher than the error rate for the standard paper-based method (p = 0.055).

#### Missing values - Lowest rates for missing values for tablet PC and netbook

**Figure 3:**
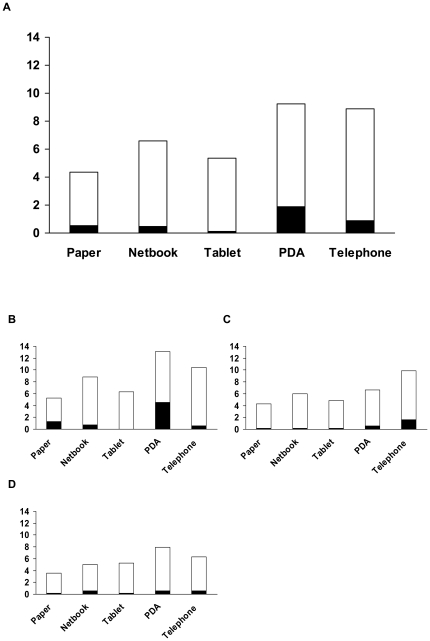
Missing values. The overall error rate (%) was defined as error per 100 fields and included the missing values. In the graph above the overall error rate is split into error rate, defined as incorrect entries per 100 fields (white), and missing rate, defined as missing values per 100 fields (black). The missing rates for the 1st, 2nd and 3rd week are presented in the three smaller graphs.

The highest rate of missing values (defined as missing values per 100 fields) of 1.86% (31/1,665) for any type of question for the entire study period was measured for the PDA, which is due to the partial deletion of a record in the first week (21 missing values for one record). With 0.90% (15/1,665) the missing rate for the telephone interview was higher than the missing rate of 0.54% (9/1,665) measured for the standard paper-based method. Compared to the standard method a similar rate was measured for the netbook 0.48% (8/1.665). The tablet PC proved to be superior with 0.1% (2/1,443) missing values (Figure 3). In OpenClinica® the function of required fields was implemented for all field types, except for skip questions. With the netbook, tablet PC and the telephone missing values were therefore only observed for skip questions.

### Duration of the interviews and data entry

#### At the end of the study less time was needed for the interviews than at the beginning of the study. - Interviews could be conducted significantly faster with the standard CRF based method than with EDC, regardless of which device was used

In the first week the median duration was shortest (21 min) for the interviews in which the data was collected in the standard, paper-based way (table 3). The median durations of the interviews in which data was collected electronically were significantly higher. Lowest median duration of interviews using EDC methods was measured for the netbook (43 min). Compared to this method, the median duration was longer for the telephone interview (54 min), the PDA and the tablet PC (64.5 min and 66 min, respectively). Over the study period of three weeks the median duration of the interviews improved for the standard paper-based method (−19%) as well as for all EDC methods (netbook (−11.6%), tablet (−50%), PDA (−37%), and telephone (−40.7%)), with the reduction being significant for the telephone interviews (p_adjusted_ 0.034) and the interviews using the tablet PC (p_adjusted_ 0.046).

**Table 3:** Duration of the interviews.

Method	1st week		2^nd^ week		3rd week		3^rd^ week compared to 1^st^ week	Trend over study period
	Median (min)	Range* (min)	Median (min)	Range* (min)	Median (min)	Range* (min)	p**	p_adjusted_ ***
**Paper**	21	17–29	16	10–20	17	12–21	0.056	0.133
**Netbook**	43	30–66	45	24–57	38	27–50	0.078	0.168
**Tablet**	66#	33–78	45	32–75	33	23–50	0.109##	0.001
**PDA**	64.5 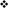	49–88	49	41–80	40	29–67	0.068	<0.0001
**Telephone**	54	35–64	40	27–48	32	22–41	0.042	<0.0001

*Minimum and maximum duration of the interviews.

**Comparison of the median duration of the interviews between the first and the last week of the study period for each device using the Wilcoxon signed-rank test.

***Mixed effect model fitting device, week, sequence and interaction terms for device/week, adjusting for random effects for the 25 combinations of fieldworker and device. P-values indicate if there was a significant reduction in duration of the interviews over the study period of three weeks.

# Median duration for three interviews using the tablet PC.

## During the first week of the study 3 of 5 scheduled interviews for the EDC method using the tablet PC were conducted. The p-value of p = 0.109 for the non-parametric test might therefore not be a reliable estimate.

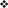
Median duration for four interviews using the PDA.

**Figure 4:**
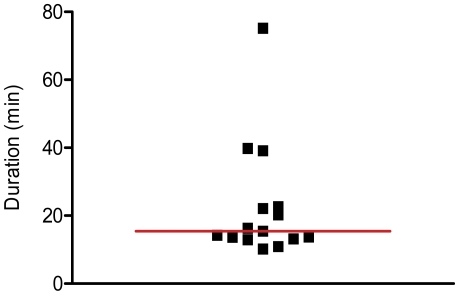
Duration of data entry per questionnaire. The figure presents the time (min) a data entry clerk needed to transcribe the data from one paper Case Report Form to an electronic record. The median time is represented by a red line.

The median time a data entry clerk needed to enter the data from one questionnaire (single data entry) was 14.5 min (Inter Quartile Range: 13.2–22.63 min) (figure 4).

After adjusting for device and week the overall error rate was not significantly associated with the duration of the interview (p = 0.33), which suggests that interviews which were conducted faster were not necessarily prone to a higher error rate.

Duration of the interviews was highly associated with the age of the interviewer (p<0.0001). Younger interviewers conducted the interviews in shorter time periods than older interviewers. However, the error rate was not associated with age (p = 0.53), but interviewers with more work experience produced lower error rates than those who were less experienced (p = 0.003).

### Costs

After the EDC introduction period of 2 weeks the estimated salary costs per correctly-entered field for EDC using the tablet were 5.8% reduced compared to the standard data capture method, which served as a reference. However, compared to the standard method the salary cost for EDC using the netbook were 5.2% increased due to the increased duration of the interview.

For the purchase of the technological equipment for EDC, netbook and tablet PC, 57% and 109% more funds had to be invested, respectively, than for the standard desktop, which was used for double entry of the data. Since EDC devices are exposed to heat, dust and humidity, and have to be transported frequently to and in the field – often on motorbikes, the purchase of ruggedized devices, which are sealed and promise to be shock-proved to a certain extend, might be recommendable, which would implement additional costs. The longer duration of EDC interviews will lead to a reduction of the number of interviews, which can be conducted per fieldworker and day. The smaller number of interviews will add to the costs for fieldwork either by increased study duration or employment of more staff and purchase of additional EDC devices. In both cases cost for transportation will be higher for EDC compared to the standard method. To ensure that fieldwork would not be compromised by defective devices, replacement devices should be available. In table 4 cost factors for GCP compliant EDC using the netbook or tablet were listed in comparison with the standard data collection method. The PDA was not included in the cost comparison, since the technology was very different to the technology which was used for the netbook and tablet.

**Table 4:** Comparison of cost estimates for standard and Electronic Data Capture (EDC) methods.

Type of costs	Standard Method	EDC using Netbook	EDC using Tablet
**Salary** *	**1.0**	1.052 **(+5.2%)**	0.942 **(−5.8%)**
	training of nurses/fieldworkers and data entry clerks	training of nurses/fieldworkers	training of nurses/fieldworkers
**Technology**	desktop	netbook **(+57%)**	tablet PC **(+109%)**
	server space	use of ruggedized devices	use of ruggedized devices
		replacement device(s)	replacement device(s)
		server space	server space
**Overhead**	office space for data entry clerks	additional costs for transportation (cars/motor-bikes)**	additional costs for transportation (cars/motor-bikes)**
	archiving paper CRFs		

*Salary costs per correctly entered field were calculated according to the formulas which are presented in the methods section. The time a data supervisor (standard method) or a data manager (EDC) would need for the synchronization of the data bases and the performance of range and consistency checks was assumed to be 5 min/questionnaire. Duration of double entry of the data of one record was calculated by multiplying the median duration for one entry by two. The values for the interview duration and error rates for the standard and EDC methods were taken from the last week of the study. The costs for netbook and tablet PC are presented as relative percent increase/decrease compared to the standard method.

**The duration of the interviews using EDC was longer compared to the standard paper-based method. The number of interviews per fieldworker and working day might therefore be reduced with EDC compared to the standard method. The costs for transportation might increase consequently.

## Discussion

A major advantage of EDC is that data become available after collection in the field without delay. This enables EDC users to monitor data collection, evaluate the study status and to review and analyse data in real-time.

So far the literature is mainly descriptive when comparing EDC to the conventional paper method. Analytic studies usually compare the transcription (CRF-to-database) error rates for two different devices such as for example a laptop and a handheld device.

This study confirms that after a short period of EDC introduction, electronic data capture can be more time effective than the standard, paper-based data capturing process followed by double entry and verification, although the duration for the interviews using the standard paper questionnaire were shorter compared to the interviews, in which the data was collected electronically.

The second advantage, which has been described in the literature, that electronic data capture is more accurate than the paper-based method could not be confirmed in this pilot study. Even though the interviewers familiarised themselves with the new data capturing methods very quickly and the overall error rate decreased considerably over time for all EDC. For two EDC methods (tablet PC and netbook) error rates approached those of the standard method, but were never lower. In addition, since the main objective of this pilot study was to test if EDC was feasible and to explore which EDC method(s) proved to be the most suitable for the next step of field studies, range and consistency checks to optimize data quality were not implemented.

It should be noted that text fields were overrepresented in the applied questionnaire due to sample size considerations. The 2010 Demographic and Health Survey (DHS) model household questionnaire [19], or the 2010 Multiple Indicator Cluster Survey (MICS) household questionnaire [20] comprised roughly 5 or 11% ‘text field’ questions, respectively, depending on household size. The most prevalent type of question in the DHS or MICS surveys was the single select type. Errors in text fields were based on exact comparisons, other than case, which might have resulted in an overestimation of the error rate for character based data, since some text field errors might have been of minor importance. For example, the misspelling of names is usually inconsequential due to unique identifiers. On the other hand this pilot study was not sufficiently powered to detect small differences in data accuracy within scales which might be important for large field studies. Direct comparison of the performance of the PDA with the remaining EDC devices is limited since a different, web based application was used for the PDA. High error rates for this device in the first week could be explained by the high proportion of missing values due to the partial loss of data. In OpenClinica® the required fields feature was implemented but not in the web based application, which might account for higher error rates for the PDA in general. The relatively poor performance of the telephone interviews is most likely due to miscommunication. Administering the questions via telephone might also have increased the interview duration since questions/answers had to be repeated more often than in face to face interviews. Interviewers improved rapidly with respect to i) the time needed to conduct the interview ii) to record the information electronically and iii) data accuracy. Since the study was designed as a pre -study for the duration of three weeks with a brief training phase, we can not judge if the interviewers had reached their highest level of performance, or if further significant improvement could be achieved. A field study would require a more intensive/longer training where a pilot phase is standard procedure. Another important consideration is whether field workers would be able to maintain their performance in the field. Comparing data accuracy for the EDC and conventional data capture process should certainly be part of follow up studies in the field, which are appropriately designed to address this question with respect to samples size and study duration.

A commonly-raised question about the feasibility of EDC in a setting like The Gambia is acceptance among field workers and whether they are capable of handling the electronic devices and software for electronic data capture. With the random selection of 6 (5 interviewers & 1 reserve) of the 12 workers nominated by the four MRC programs (Bacterial Diseases, Viral Diseases, Malaria and Nutrition), we sought a group of interviewers representative in their age, gender, work experience and performance, computer legacy, and attitudes towards EDC. Although fieldworkers were ultimately selected randomly, each programme purposely nominated three candidates and selection bias can not be ruled out completely.

At the beginning of the training session for this study, 5 out of 6 interviewers stated that EDC would be appreciated by the MRC field workers and that, since field workers have established a very good relationship with their study participants in the past, the participants would likely embrace EDC. The field workers/nurses who participated in this study were keen to learn new techniques and develop themselves further.

The majority of the interviewers would prefer the ‘bulkier’ devices, netbook and laptop, which were used for electronic data capture during the telephone interviews, to the smaller devices tablet PC and PDA for field work. That older people find it more satisfactory to use the ‘bulkier’ devices with a keyboard rather than the smaller pen-based devices has already been described in the literature [21]. The display of the QWERTY keyboard on the screen of a palm-size computer results in each key being very small. This slows performance [22] and may also give rise to legibility problems with difficulty in discriminating between some letters such as ‘u’ and ‘v’. The limited screen space also results in a lack of gaps between adjacent keys. This necessitates fine motor control by those making entries. The space available on the screen is very small so that the keyboard often requires the use of special function keys to access certain numeric and punctuation characters. It has long been known that moded styles of interaction can be confusing for users [23], [24]. All interviewers in our study were already familiar with the standard keyboard and had at least two years MRC work experience; each also accessed their MRC e-mail account at least once a week. The familiarity with the keyboard enabled the interviewers to enter data within a shorter time period after training compared to devices which required use of a pen. Another reason given for preferring the bulkier devices was that the larger screen allowed working with larger font sizes. Some of the field workers, especially the older ones, had problems with their eyesight. During the study interviewers would only use their glasses when the font size became so small that they could not cope otherwise. In the Gambian setting reluctance to the use of glasses is common. While two fieldworkers had problems reading text on the PDA and the tablet PC, prompting one of them to use his glasses, no such problems occurred with the netbook or the laptop. Nevertheless, in this study the use of the pen/touch screen improved rapidly for all fieldworkers , so that in the final week the duration of the interviews using OpenClinica® as the front end, which did not depend on the network connection, was in a similar range, regardless if a keyboard or a pen/touch screen was used.

Studies of performance with normal size keyboards have shown that although older people are slower in key tapping and in selecting the key to tap [25], especially if they are unskilled typists [26], there is no loss of accuracy. Given sufficient experience with the task there is evidence that older people may be able to use compensatory strategies to maintain their performance [27]. This study lends support to this statement, since accuracy was not associated with age. Speed was highly associated with age, however. Younger interviewers conducted the interviews in less time than their older colleagues. As one would expect, interviewers with more experience in fieldwork were superior with respect to accuracy than those with less fieldwork experience.

Despite the availability of diverse data management software applications, it was beyond the scope of the study to test each implementation for compatibility with specific EDC tools. The data management unit at the MRC routinely supports 10 simultaneous clinical (phase I or II) and research trials; to be of practical relevance, EDC tools must be capable of supporting the commonly used software platforms. The open source, clinical trials software for EDC and clinical data management OpenClinica® was used for three of the four EDC methods, since this clinical informatics system has been designed to support GCP standards. Unfortunately an OpenClinica® version for PDAs was not available and a web based system was established. The PDA is at a disadvantage in this regard as coding a bespoke solution or integrating an open source/commercial application is time consuming and potentially expensive if commercial software is used.

At the MRC in the Gambia various types of field based epidemiological studies are conducted apart from clinical trials. For those studies alternative data capturing methods such as improved web based systems or telephone interviews in combination with EDC, or standard data collection on paper followed by optical character recognition (OCR) might be more appropriate or more cost effective.

Obvious rules, which apply for telephone interviews in general and can be found in many standard epidemiological text books, will be valid for telephone interviews in which data is collected electronically as well. For short follow up studies, in which a physical examination of the interviewee is not needed, telephone interviews might be a quick and cheap alternative to sending a field worker to the field, especially since the Gambia enjoys excellent mobile phone coverage.

A South African group recently described a web based system using standard mobile phones for electronic data capture [28]. The questionnaire was developed in a word processor and sent to a standard mobile phone, which was used to collect the data during a survey. The data was stored as a text file and whenever the network connection was available, the encrypted information was sent via SMS to the host computer. The automated data upload from the mobile phone to the server reduces data loss due to damage , theft or loss of the device. Mobile phones have the advantage, that they are available, cheap, relatively robust and less attractive targets for theft compared to the more expensive devices tablet PC and netbooks. The downside of this method is, that techincal problems with data upload and download [29], electrical interference with telephone lines [30] and considerable problems with the synchronization process [31] have been encountered in the past.

In May 2010, a new web services framework became available in OpenClinica®, which supports mobile devices as a means of data collection for clinical trials [32].

For surveys, with a high percentage of tick questions can be developed, the OCR might be the appropriate choice.

EDC gives scope for applications in combination with additional functions such as GPS for identification and retracing of households, probability sampling for household surveys, which results in statistically valid samples, rapid aggregation, analyses, and availabitlity of preliminary results within days of completing the field work [33], or barcode reading to identify study subjects or samples.

EDC promises to expedite the availability of accurate data for the research program. However, there are considerable risks in achieving this goal. Pens might be lost, devices might be defective, broken or stolen. Especially in a setting like the Gambia devices are exposed to dust and humidity, and are transported on motorbikes during the day to day routine. Malfunction or loss of the device might imply that the data won't be collected according to schedule, or that data which have been already collected might be lost or have to be collected again. This will rapidly increase the time spent on gathering such information and error rates are likely to increase. For this situations a rescue plan should be established, which might involve provision of a replacement device, which will be readily available within a short period of time. Longer study duration and replacement of devices are associated with increased costs.

### Recommendations

A good study design is important and helps to identify the variables, which are essential to meet the objective of a study. Focused and accurate data collection will increase study efficiency.

When properly designed, EDC solutions can offer a convenient, cost-effective approach for data entry, management data and reporting. Major challenges for EDC are the potential loss of the source documents, disruption of the data processing due to inappropriate human operation or inadequate maintenance of the computerized system and the need for suitable storage and maintenance of electronically captured data in a repository to make data readily available.

Before implementation in the field in a wide range of studies, one should use the results from this pilot study to develop EDC further and test this improved EDC method in the field. During the field study other factors like battery life, functionality of the devices under field conditions (dust, humidity, and transport on motorbikes) will have to be assessed. It will be essential to compare the results to the conventional paper format with respect to speed, error rate and costs. A pilot study or an extended training phase before the start of the actual study would be recommendable. This will help to identify potential major difficulties, and will give the fieldworker the opportunity to learn how to troubleshot common problems.

Abandoning paper-based systems will require not only new technologies, but additionally new work processes. This pilot study shows, that single select questions have much lower error rates compared to free text and date fields. In order to archive highly accurate data with EDC, questionnaires should be designed to maximise the use of single select or multiple choice questions and minimise the number of text and date fields. In this pilot study date fields were associated with significantly high error rates for two devices using OpenClinica® data capture compared to the web based system (PDA). OpenClinica® provides a calender function for entering dates. If the interviewer chooses to use this function, she/he markes the date on a small calender, which pops up next to the date field. The interviewers, who used this function had significantly increased error rates in the date fields, whereas the error rates for other fields such a text or single select fields for these interviewers were not increased. Dates were usually shifted one or two months or years forwards or backwards, which indicates that the interviewer missed the correct field, when choosing the date. Since the calender function seemed to be associated with increased error rates, alternative methods such as separate fields for day, month and year, should be implemented.

The introduction of EDC requires programming and data base development capacity. At the time the study was conducted OpenClinica® had no branching or skip logic implemented, but development and implementation of branching logic is crucial to reduce the error rate for missing fields. The skip logic is now available with the new OpenClinica® 3.1 beta version [34].

The importance of the user friendliness and quality of training materials for an EDC system was recently described to outweigh the features and functionality for small scale clinical trials [35]. Interviewers should receive thorough training and retraining to improve accuracy. A practical approach might be to train a group of capable field workers/nurses, who could supervise other field workers and give support during field work. Field workers should have access to rapid trouble shooting. For trouble shooting one could implement a stepwise procedure. An interviewer might be equipped with a manual guiding the interviewer to solve some common problems by him/herself. He/she should have access to a more experienced colleague, who might be able to assist when the interviewer can't solve the problem. For more difficult problems a data base developer/programmer should be available for in depth troubleshooting.

To improve data quality it might be an advantage to offer ophthalmological examinations to the interviewers and to emphasize the benefits of wearing glasses in general and how it would positively influence the day to day work.

This study was conducted under controlled conditions, in which interviewers were supported by experienced data base programmers/developers and OpenClinica® experts, without any delay, whenever a problem arose. Aspects such as battery life, longevity of the electronic devices under field conditions (dust, humidity and frequent transport on motorbikes) and synchronization of data bases have not been addressed. The next step would be to further improve the electronic data collection method (optimized ‘work process’ for EDC with respect to questionnaire and data base design, introduce branching logic in OpenClinica®, chose the optimal device for the field work) and use an EDC method in parallel to the standard data collection method in an actual field study. Electronic devices should be selected with care with respect to brightness, size of screen, battery life and performance of the touch screen. Since EDC devices would be exposed to heat, dust and humidity, and have to be transported frequently in the field, ruggedized EDC devices might prove superior for the day to day routine. If the initial higher investment in such devices pays off in the end and guarantees fieldwork to run smoothly, might be worthwhile to be investigated in the future.

### Conclusions

This pilot study shows that MRC field workers and nurses would be able to handle electronic devices and software for EDC, with appropriate training, retraining and adequate support during their field work. The tablet PC and the netbook with OpenClinica® as the front end performed better than the PDA and the telephone interview. Interviewers preferred the ‘bulkier’ netbook devices with a keyboard compared to the tablet PC/pen/touchpad combination for data entry.

In summary, if EDC is well designed and introduced with care, and work processes are adjusted to EDC, it will become a more time effective, potentially more accurate, and therefore cost effective method than the standard paper-based data collection method.

## Supporting Information

File S1 **Specifications of the electronic devices.**
(DOC)

File S2 **Access data base.** The Access data base holds the original data, as they were generated for reference, the validated and verified data for the standard paper-based method, and all four EDC data sets. Each data set comprises an adult and a child data sheet. It has to be stressed that the data for this study was generated, which means that all personal information is fictive and any resemblance with living persons occurs by chance.
(BZ2)
