# Supplementary material for: Comparison of Electronic Data Capture (EDC) with the Standard Data Capture Method for Clinical Trial Data
Source: PLoS One. 2011 Sep 23;6(9):e25348. doi: 10.1371/journal.pone.0025348 (PMC3179496; doi:10.1371/journal.pone.0025348)
Supplement: File S1 — Specifications of the electronic devices. (DOC) [file pone.0025348.s001.doc]

**Supporting Information. File S1**

**__________________________________________________________________________________**

**Specifications of the electronic devices**

**Netbook:** Lenovo ThinkPad SL500 2746 with 2.2 GHz Intel Core 2 Duo T6670 processor, 15.4’’ widescreen in TFT active matrix display, Windows XP Professional operating system, weight of 2.9 kg, 4 hours battery life (£ 535)

**Tablet PC:** Samsung Q1 Ultra with 800 MHz Intel A 110 processor, 7’’ touch screen in TFT active matrix display (1024 x 600), Microsoft Window SP Tablet PC Edition operating system, weight of 0.69 kg and 10 hours battery life (£ 710)

**PDA:** Hewlett-Packard iPAQ 214 Enterprise Handheld with 624 MHz Marvell PXA310 processor , wireless and Bluetooth® with Enhanced Data Rate (EDR) and 4’’ touch screen in TFT (640 x 480), Windows Mobile® 6 Classic operating system and a weight of 190g. (£225.92)
